# Editorial: Fifty Shades of Grey: Exploring the Dark Sides of Leadership and Followership

**DOI:** 10.3389/fpsyg.2018.01877

**Published:** 2018-10-05

**Authors:** Susanne Braun, Ronit Kark, Barbara Wisse

**Affiliations:** ^1^Durham University Business School, Durham University, Durham, United Kingdom; ^2^Department of Psychology, Bar Ilan University, Ramat Gan, Israel; ^3^Department of Psychology, University of Groningen, Groningen, Netherlands

**Keywords:** abusive supervision, dark side, destructive leadership, followership, Machiavellianism, narcissism, psychopathy

“I could stand in the middle of Fifth Avenue and shoot somebody and I wouldn't lose any voters. Okay. It's like incredible!” Donald J. Trump, President of the United States, in January 2016 at a campaign rally in Iowa.

In light of corporate and political turmoil and subsequent questions raised about leaders' dark sides, this Research Topic is particularly timely. We set out to contribute to theoretical, empirical and methodological advancements, focusing on dark sides of personality, processes, and perceptions, and how they relate to leader-follower relationships. Studies of the dark side of leadership follow a long-standing tradition (Conger, 1990), and initially focused mainly on negative leader traits such as narcissism (Braun, 2017) and leader behaviors such as abusive supervision (Hogan and Kaiser, 2005; Tepper, 2007; Schyns and Schilling, 2013; Tepper et al., 2017). The particular potential for toxicity to unfold at the intersections of leadership and followership has been noted (Padilla et al., 2007), yet research into this domain remains largely underdeveloped. While followership theories receive increasing attention (Uhl-Bien et al., 2014), the potential dark sides of followership or followers' impact on dark-side leaders remain unclear. Deviating from the unidimensional view that leaders are omnipotent and to be blamed for negative outcomes, we seek to place emphasis on the different “shades” of dark leadership by focusing on how dark leadership can be explained by taking leaders, followers, and their interaction in specific contexts into account.

In line with the purpose to explore the intersections between dark-side leadership and followership, we saw three main themes emerging from the articles published in this Research Topic. The first theme revolves around leader traits and behaviors. It focuses on questions such as what makes a “dark-side” leader and what “dark-side” leaders do. The second theme accounts for the interaction between leaders' and followers' characteristics, and zooms in on the extent to which this interaction may affect the negative impact of “dark-side” leadership or followership. Finally, the articles also reflect novel ideas, extensions and integration of current theories at the interface between leadership and followership.

## Leader traits and behaviors

The conceptual paper by de Vries reviewed personality traits and their links with dark leadership styles. The Three Nightmare Traits (TNT), leaders' dishonesty, disagreeableness, and carelessness, were found to be aligned with low honesty-humility, agreeableness, and conscientiousness. Using a Situation-Trait-Outcome-Activation (STOA) model the author argued that specific situations should attract TNT leaders, activate their dark-side traits, and result in (mainly but not exclusively) negative outcomes in relation to the recognition, perception, and attribution of leadership.

In addition, three of the published articles gave primary attention to the question of what dark-side leaders do and how they affect followers at work and in terms of their personal lives. This widens our scope of leadership behaviors that are perceived as negative, and allows us to explore more discrete types of negative behaviors and their outcomes.

Three different types of destructive leadership and their effects on follower outcomes were assessed in an experiment and a field study by Schmid et al. Differentiating between distinct types of negative leadership their research focuses on follower-directed (abusive supervision), organization-directed, and self-interested (exploitative) destructive behaviors. All three forms of dark-side leader behaviors predicted followers' negative affect. However, abusive supervision elicited the highest levels of fear. In relation to turnover intentions, exploitative leadership and abusive supervision affected calculative and immediate turnover intentions similarly.

Nauman et al. extended the research to explore how dark-side leadership affects the private sphere of life of the employees. They assessed despotic leadership (i.e., tendencies toward authoritarian and dominant behavior in pursuit of self-interest, self-aggrandizement, and exploitation of others) and its negative effects, which the authors hypothesized would transcend from the workplace to subordinates' personal lives (increased emotional exhaustion and work-family conflict, and decreased life satisfaction). The results confirmed their hypotheses. They show that negative forms of leadership can also affect our personal lives, homes and families and opens up a new field of research at the work-life interface. The work also connects with our second theme, the interplay between traits of leaders and followers. In this study, followers' anxiety increased the negative impact of despotic leadership.

Schyns et al. extended the perspective from dark-side leader behaviors to follower perceptions and attributions of these behaviors. Comparing different levels of abusive behavior (constructive leadership, laissez-faire leadership, mild to strong abuse), they analyzed follower perceptions of abusive supervision and follower attributions as moderators. The three-study series employed manipulations of leaders' abusive behaviors and established attributions of the leaders' intentionality in the behavior and the level of his/her control as moderators. Relationships between abusive supervision perceptions and outcome variables (loyalty, turnover, and voice) were largely buffered by the attribution of leader intentionality. In Study 3, a survey of abusive supervision perceptions, however, control attributions strengthened the relationships with loyalty and voice.

## The interplay between traits of leader and follower

Three articles in this Research Topic provided a largely new angle. They considered the relevance of follower traits when confronted with dark-side leadership, but also followers' own dark-side traits.

Looking at leader narcissism, Nevicka et al. analyzed the interface between self-absorbed, entitled narcissistic leaders and insecure follower, who make “easy targets” for narcissists. The authors conducted two field studies. Followers with low self-esteem and low core self-evaluations perceived narcissistic leaders as more abusive than those with high self-esteem or high core self-evaluations. Abusive supervision perceptions in turn related to lower follower performance and higher experiences of burnout, pointing to risks of leader narcissism for vulnerable followers.

Barelds et al. also studied followers' self-esteem, but in terms of how it affected the relationship between leaders' psychopathy and their self-serving behaviors. The authors first conducted an experimental study, in which they manipulated follower self-esteem, measured leader psychopathy, and assessed their combined effects on leader self-serving behavior using an ultimatum game. They also conducted a multi-source field study using questionnaires to assess leader psychopathy, follower self-esteem, and perceived leader self-serving behavior. Across both studies they found that leader psychopathy was positively related to leader self-serving behaviors, but only when their followers had low rather than high self-esteem. Again, these findings show that that the degree to which dark-side traits of leaders are reflected in their behavior depends on the characteristics of their followers. Follower characteristics can mitigate the negative impact of dark-side leadership.

However, not only leaders' dark-side traits pose risks to organizations; followers' dark-side traits may do the same. Belschak et al. studied ethical leadership as a potential remedy for negative behaviors of Machiavellian followers. Followers with high Machiavellianism are goal-driven to the extent that they use all possible means to achieve desired ends. Machiavellianism predicted reduced helping behavior and increased knowledge hiding and emotional manipulation, but only when ethical leadership was low. That is, ethical leadership served as a buffer of the negative outcomes of dark-side followership.

## Novel extension of the theory and integration

Two articles challenged current theoretical thinking at the interface of leadership and followership. One article focused on the conditions under which leaders' positive efforts can in fact backfire, and the other one addressed the relevance of negative followership theories at the group level.

Kipfelsberger and Kark developed a theoretical model to explain the conditions under which leaders' meaning making efforts, despite their good intentions, can “kill” followers' experiences of meaningfulness at work. The authors applied a wide angle taking into account leaders' characteristics, followers' characteristics and the context. They argued that leaders harm followers' work meaningfulness when followers' experiences of coherence, purpose or significance of work are diminished. The six conditions that can affect the reduction of followers' sense of meaningfulness included in the model capture leaders' personality traits, leaders' behaviors, the relationship between leader and follower, followers' attributions, followers' characteristics, and job design. The negative consequences of diminished meaningfulness comprise cynicism, disengagement, and decreased well-being.

Leung and Sy extended the established construct of implicit followership theories to the group level showing that Golem effects can occur as a consequence of negative beliefs held within teams. Golem effects capture a special case of self-fulfilling prophecies, the idea that negative performance expectations result in low performance. The authors studied naturally occurring Golem effects in the form of negative implicit followership theories, specifically incompetency schemas that are shared within groups. Results confirmed showed groups who shared negative group-level Implicit Followership Theories (GIFTs) affected follower performance negatively through decreased self-efficacy and effort.

## Conclusion

We see the extension and integration of leadership and followership theories in the dark-side realm as one of the major contributions of this Research Topic. The work presented places particular emphasis on the role that followers can play in dark-side leadership, whether through their own traits, implicit theories or attributions. We also see the importance of the context as one major aspect for further investigations. Future research should add to the understanding of how leaders, followers, their relationships and the context interact within the dynamic of dark sides in organizations. Moreover, future research can look into how negative leadership affects different life spheres of the followers, as well as of the leaders themselves, We see particular strengths of the empirical papers presented here in their methodological rigor, including experimental as well as survey data, gathered from multiple sources and in multiple-study series. Better understanding the dark sides of leadership and followership is, so we believe, timely. Future research may decipher more unique and discrete types of dark leadership and followership, focus on toxic relationships and their consequences, and find ways to reduce the harmful effects. In other words, there can be at least “50 shades of gray” in dark-side leadership.

## Author contributions

All authors listed have made a substantial, direct and intellectual contribution to the work, and approved it for publication.

### Conflict of interest statement

The authors declare that the research was conducted in the absence of any commercial or financial relationships that could be construed as a potential conflict of interest.
